# Congenital long QT syndrome

**DOI:** 10.1186/1750-1172-3-18

**Published:** 2008-07-07

**Authors:** Lia Crotti, Giuseppe Celano, Federica Dagradi, Peter J Schwartz

**Affiliations:** 1 Section of Cardiology, Department of Lung, Blood and Heart, University of Pavia, Pavia, Italy; 2 Department of Cardiology, IRCCS Fondazione Policlinico S. Matteo, Pavia, Italy; 3 Molecular Cardiology Laboratory, IRCCS Fondazione Policlinico S. Matteo, Pavia, Italy; 4 Laboratory of Cardiovascular Genetics, IRCCS Istituto Auxologico, Milan, Italy; 5 Department of Medicine, University of Stellenbosch, South Africa; 6 Cardiovascular Genetics Laboratory, Hatter Institute for Cardiovascular Research, Department of Medicine, University of Cape Town, South Africa

## Abstract

Congenital long QT syndrome (LQTS) is a hereditary cardiac disease characterized by a prolongation of the QT interval at basal ECG and by a high risk of life-threatening arrhythmias. Disease prevalence is estimated at close to 1 in 2,500 live births.

The two cardinal manifestations of LQTS are syncopal episodes, that may lead to cardiac arrest and sudden cardiac death, and electrocardiographic abnormalities, including prolongation of the QT interval and T wave abnormalities. The genetic basis of the disease was identified in the mid-nineties and all the LQTS genes identified so far encode cardiac ion channel subunits or proteins involved in modulating ionic currents. Mutations in these genes (*KCNQ1*, *KCNH2*, *KCNE1, KCNE2*, *CACNA1c*, *CAV3*, *SCN5A*, *SCN4B*) cause the disease by prolonging the duration of the action potential. The most prevalent LQTS variant (LQT1) is caused by mutations in the *KCNQ1 *gene, with approximately half of the genotyped patients carrying *KCNQ1 *mutations.

Given the characteristic features of LQTS, the typical cases present no diagnostic difficulties for physicians aware of the disease. However, borderline cases are more complex and require the evaluation of various electrocardiographic, clinical, and familial findings, as proposed in specific diagnostic criteria. Additionally, molecular screening is now part of the diagnostic process.

Treatment should always begin with **β**-blockers, unless there are valid contraindications. If the patient has one more syncope despite a full dose **β**-blockade, left cardiac sympathetic denervation (LCSD) should be performed without hesitation and implantable cardioverter defibrillator (ICD) therapy should be considered with the final decision being based on the individual patient characteristics (age, sex, clinical history, genetic subgroup including mutation-specific features in some cases, presence of ECG signs – including 24-hour Holter recordings – indicating high electrical instability).

The prognosis of the disease is usually good in patients that are correctly diagnosed and treated. However, there are a few exceptions: patients with Timothy syndrome, patients with Jervell Lange-Nielsen syndrome carrying *KCNQ1 *mutations and LQT3 patients with 2:1 atrio-ventricular block and very early occurrence of cardiac arrhythmias.

## Background

The congenital long QT syndrome (LQTS) is a relatively uncommon but important clinical disorder. Since 1975 [1] it includes under the unifying name of "Long QT syndrome" two hereditary variants. One is associated with deafness [2,3] and one is not [4,5]; they are referred to as the Jervell and Lange-Nielsen syndrome (J-LN) and as the Romano-Ward syndrome (R-W), respectively. Long-QT syndrome has been subdivided into types based on the gene in which causative mutations occur. The most prevalent forms are LQT1 and LQT2 (due to mutations in potassium channels), and LQT3 (due to a sodium channel mutation).

The clinical manifestations of the disease are rather dramatic as they involve syncopal episodes, which often result in cardiac arrest and sudden death and usually occur in conditions of either physical or emotional stress in otherwise healthy young individuals, mostly children and teenagers. The high lethality among symptomatic and untreated patients in the presence of very effective therapies makes unacceptable the existence of symptomatic and undiagnosed patients. The genetic findings of the last 15 years have made of LQTS a unique paradigm for genetically mediated sudden cardiac death that allows to correlate genotype and phenotype, and provides a direct bridge between molecular biology and clinical cardiology.

## Epidemiology

Initially considered as a very rare condition, already in 1975 [1] we suggested that LQTS "could be more unrecognized than rare". When coming, however, to actual numbers everything seemed to go and the prevalence was assumed to be anywhere between 1/5,000 [6] to 1/20,000 [7], with most investigators settling for 1/10,000 [8]. Importantly, none of these estimates was based on actual data.

The first data-driven indication of the prevalence of LQTS is coming from the largest prospective study of neonatal electrocardiography ever performed [9]. An electrocardiogam (ECG) was recorded in 44,596 infants at 3–4 weeks of age. Among them, 1.4% had a corrected QT (QTc) interval between 440 and 469 ms and 0.7/1,000 had a QTc ≥ 470 ms, regarded as markedly prolonged by the European Task Force on Neonatal Electrocardiography [10]. In the latter group (n = 31), more than 90% of infants underwent molecular screening and LQTS disease-causing mutations were found in 13 of 28 (46%) [9]. As almost 50% of the infants with QTc ≥ 470 ms (0.7/1,000) are affected by LQTS, and as at least some (number being currently defined by extensive molecular screening) of the infants with QTc between 440 and 469 ms are also likely to be affected, it follows that the prevalence of LQTS must be close to 1/2,500 at least. This is probably a bit of an underestimate because we have postulated first [11] and demonstrated later [12,13] that there is a significant number of silent mutation carriers (QTc < 440 ms) that actually ranges between 10% and 36% according to genotype. For the first time the prevalence of a cardiac disease of genetic origin has been quantified on the basis of actual data.

## Clinical description

### Romano-Ward syndrome

The two cardinal manifestations of LQTS are syncopal episodes and electrocardiographic abnormalities.

#### Cardiac events and their relation to genotype

The syncopal episodes are due to Torsade-de-Pointes (TdP), a polymorphic ventricular tachycardia with a characteristic twist of the QRS complex around the isoelectric baseline, often degenerating into ventricular fibrillation. TdP or ventricular fibrillation can initiate without changes in heart rate and without specific sequences such as "short-long-short" interval, even though long pauses in LQTS patients increase the probability of TdP [14].

While it had been known for quite sometime that although most patients would develop their symptoms under stress, it was also known that in a minority of cases these life-threatening cardiac events could occur at rest. The reason(s) for these different patterns remained obscure until molecular biology allowed to distinguish between different genotypes. As predicted by their impairment on the I_Ks _current (essential for QT shortening during increases in heart rate), most of the events of LQT1 patients occur during exercise or stress [15]. Conversely, most of the events of LQT2 patients occur during emotional stress such as auditory stimuli (sudden noises and telephone ringing, especially while at rest) while for LQT3 patients they occur during sleep or at rest [15].

A higher risk has been reported in the post-partum period [16]. Even here genotype is important because risk is higher for LQT2 than for LQT1 patients [17,18]. In our opinion, the highest risk for LQT2 women is at least in part related to sleep disruption; accordingly, we recommend not only to continue with full dose of **β**-blockers but we consider important that husbands contribute to feed the infants nighttime thus allowing a fair amount of uninterrupted sleep for their LQT2 wives.

#### Electrocardiographic aspects

**Figure 1:**
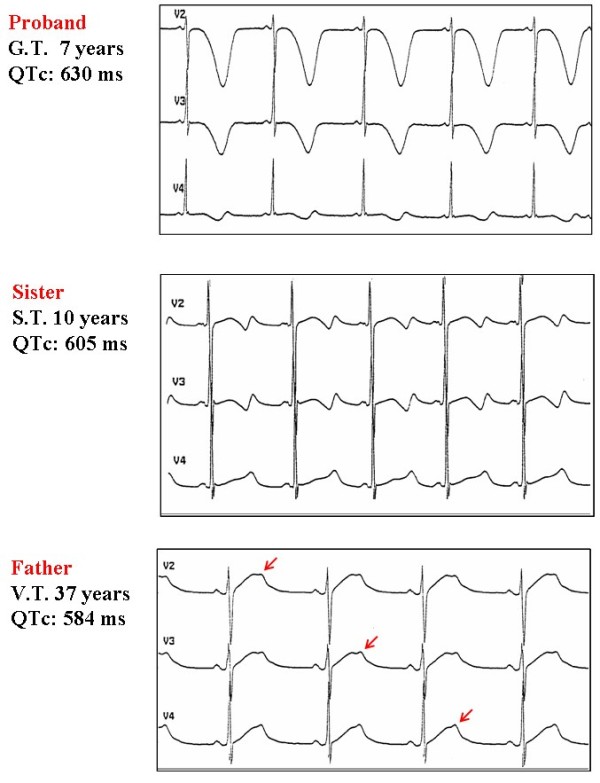
**Different T wave morphologies in affected members of the same family**. The proband had a documented cardiac arrest as first manifestation of LQTS. The basal ECG shows deep negative T waves in the precordial leads and a very prolonged QTc. His sister is still asymptomatic with typical bi-phasic T waves. His father, with notched T waves and a QTc 584 ms, has had 2 syncopal episodes. The arrows point to examples of notched T wave. *[Reprinted from: Schwartz PJ, Priori SG, Napolitano C: The long QT syndrome. In: CARDIAC ELECTROPHYSIOLOGY. FROM CELL TO BEDSIDE. III EDITION (Zipes DP and Jalife J, Eds.) WB Saunders Co., Philadelphia, pp. 597–615, 2000 – with permission from Elsevier]*.

The bizarre electrocardiogram of many LQTS patients should be easily recognized (Fig. 1). Clearly, there is much more than a mere prolongation of ventricular repolarization. The T wave has several morphologic patterns easily recognizable on the basis of clinical experience. They are difficult to quantify but very useful for diagnosis.

### QT interval duration

The Bazett's correction for heart rate remains a very useful clinical tool despite unrelenting criticism. At slow and fast rates it is important to be aware of hyper- and of under-correction. Traditionally, QTc values in excess of 440 ms are considered prolonged; however, values up to 460 ms may still be normal among females [19]. The longer QT values present among women [19] become evident only after puberty but are absent at birth [20], suggesting a role for hormonal changes. Exceptions exist and syncopal episodes occur also in patients with modest QT prolongation and even with a normal QT interval, but, in general, longer the QT greater is the risk for malignant arrhythmias and multiple evidence indicates that when QTc exceeds 500–550 ms there is a definite increase in risk [13,21].

The initial and understandable concept that QT prolongation was the essential cornerstone of LQTS was challenged by Schwartz in the 80s [11]. Theoretical considerations led him in 1980 and 1985 [11,22] to propose that some patients might be affected by LQTS and, nonetheless, have a normal QT interval on the surface electrocardiogram. The validity of this unorthodox concept has been proven by the existence of mutation carriers with a normal QT interval [12,13,23], as a consequence of low penetrance. This concept has important practical and medico-legal implications because, for example, it no longer allows a cardiologist to state that a sibling of an affected patient with a normal QTc "is definitely not affected by LQTS".

### T wave morphology

In LQTS not only is the duration of repolarization that is altered, but also its morphology. The T wave is often biphasic or notched (Fig. 1), suggesting regional differences in the time course of ventricular repolarization. These abnormalities are particularly evident in the precordial leads and contribute to the diagnosis of LQTS; they often are more immediately striking than the sheer prolongation of the QT interval.

### T wave alternans

**Figure 2:**
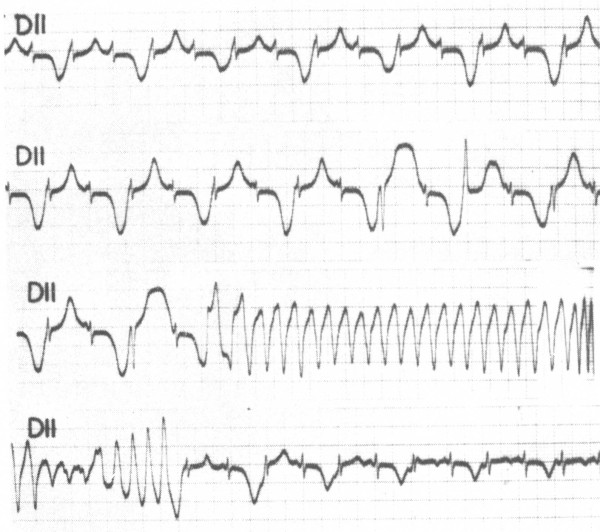
**5-year-old patient affected by LQTS with congenital deafness**. Tracing recorded during a syncopal episode. T wave alternans precedes the onset of torsade de pointes. In this case, it is also evident that TdP is not preceded by a pause. *(From C. Pernot: Le syndrome cardio-auditif de Jervell et Lange-Nielsen. Aspects electrocardiographiques. Proc Ass Europ Paediat Cardiol 1972, 8:28–36)*.

Beat-to-beat alternation of the T wave, in polarity or amplitude, may be present at rest for brief moments but most commonly appears during emotional or physical stresses and may precede TdP (Fig. 2). It is a marker of major electrical instability and it identifies patients at particularly high risk in whom reassessment of therapy should be prompted.

### Sinus pauses

Several LQTS patients have sudden pauses in sinus rhythm exceeding 1.2 seconds that are not related to sinus arrhythmia [11] and may contribute to the initiation of arrhythmias in LQTS patients. Their occurrence in LQT3 patients represents an important warning signal, which requires reinforcing safety measures.

### Heart rate and its reflex control

In 1975 Schwartz *et al*. [1] called attention to the presence of a lower than normal heart rate in many patients, a phenomenon particularly striking in children. During exercise, several LQTS patients reach a heart rate level lower than that achieved by healthy controls matched by age and sex.

**Figure 3:**
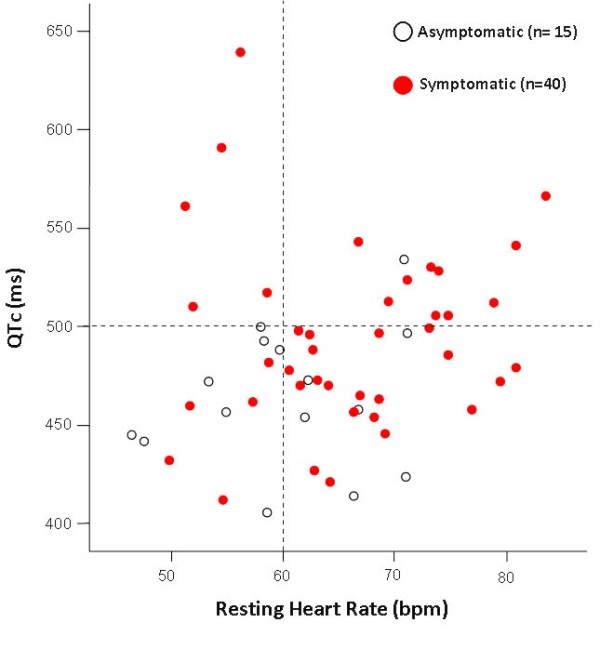
**Differential risk for arrhythmic events among Mutation Carriers (MCs) according to resting heart rate off- βB and QTc**. The dashed horizontal line represents the predefined cut-off for QTc (≤ or > 500 ms) whereas the dashed vertical line corresponds to the first tertile (≤ 60 bpm) of the heart rate values distribution. It is evident that in the group of patients with a QTc ≤ 500 ms those the first tertile (heart rate ≤ 60 bpm) were less frequently symptomatic compared to MCs in the other two tertiles (OR 0.19, 95%CI 0.04–0.79, p = 0.023). This figure also provides evidence that a QTc > 500 ms represents a more severe arrhythmogenic substrate in our population based on the fact that 15 of 16 (94%) MCs with a QTc > 500 ms were symptomatic. *(Reprinted from ref*. [25]* with permission from Lippincott Williams and Wilkins)*.

Recently, Brink *et al*. [24] and Schwartz *et al*. [25] in a large South African LQT1 founder population, in which all the affected members carry the *KCNQ1*-A341V mutation, provided the novel evidence that faster basal heart rates and brisk autonomic responses are associated with a greater probability of being symptomatic. This likely depends on the fact that LQT1 patients have an impaired ability to shorten their QT interval during heart rate increases because of the mutation-dependent impairment in I_Ks_, the current essential for QT adaptation. Whereas among patients with a major arrhythmogenic substrate (QTc > 500 ms) heart rate is rather unimportant, among patients with a less severe arrhythmogenic substrate (QTc ≤ 500 ms) those in the lower tertile of heart rate are more frequently asymptomatic (Fig. 3). Furthermore, relatively low values of baroreflex sensitivity – an index of the ability to respond with brisk increases in either vagal or sympathetic activity – were found to be associated with a reduced probability of being symptomatic. Indeed, the lack of QT shortening during sudden heart rate increases favors the R-on-T phenomenon and initiation of ventricular tachycardia/ventricular fibrillation, while sudden pauses elicit early afterdepolarizations in LQTS patients that can trigger TdP. Blunted autonomic responses, revealed by relatively low values of baroreflex sensitivity, imply a reduced ability to change heart rate suddenly which appears to be a protective mechanism for LQT1 patients.

### Jervell and Lange-Nielsen syndrome

The Jervell and Lange-Nielsen (J-LN) syndrome is the recessive variant of long QT syndrome, due to the presence of two homozygous or compound heterozygous mutations on either the *KCNQ1 *or *KCNE1 *genes [3,26,27].

**Figure 4:**
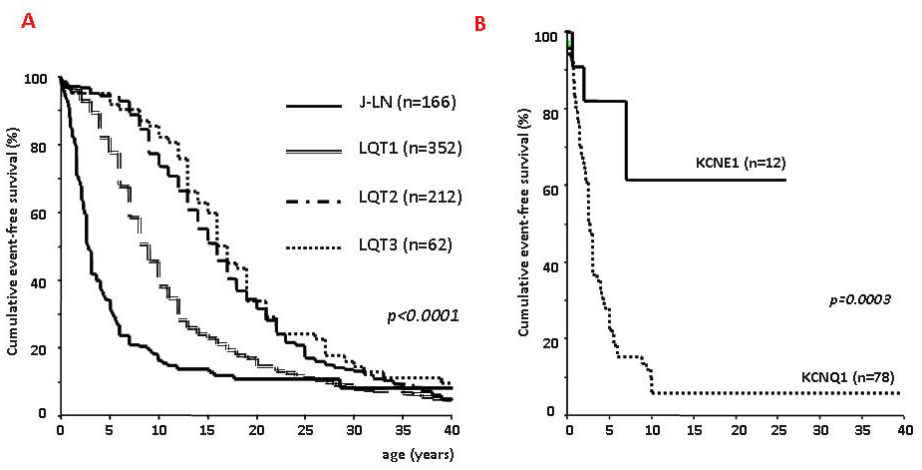
(**A) Kaplan-Meier curves of event-free survival comparing JLN patients vs LQT1, LQT2 and LQT3 symptomatic patients.***(Modified from ref*. [3]* with permission from Lippincott Williams and Wilkins)*. (B) Kaplan-Meier curve of event-free survival in JLN patients with mutations in *KCNQ1 *or *KCNE1 *genes. (*Reprinted from ref*. [3]* with permission from Lippincott Williams and Wilkins*).

At first glance, the only clinical difference between the two variants (J-LN and R-W variants) lies in the fact that J-LN patients suffer also from congenital deafness. However, a large cooperative study providing detailed clinical information on 187 J-LN patients has allowed the recognition of clear differences versus the other types of LQTS including LQT1, the variant which shares with J-LN an impairment in the I_Ks _current. The J-LN is, with the possible exceptions of the very rare forms of LQTS with congenital atrioventricular (AV) block and with syndactyly, the most severe of the major variants of LQTS. Almost 90% of the patients have cardiac events, 50% become symptomatic by age of 3 years, their average QTc is markedly prolonged (557± 65 ms), and they become symptomatic much earlier than any other major genetic subgroup of LQTS (Fig. 4A). Within the group of J-LN patients it has been possible to identify subgroups at lower risk, namely those with a QTc < 500 ms and those without syncope in the first year of life [3]. Even though the clinical diagnosis of J-LN is rather straightforward, it is important to genotype all these patients because it has been shown by Schwartz *et al*. [3] that the smaller group with *KCNE1 *mutations has a markedly less severe clinical course than that with mutations on *KCNQ1 *(Fig. 4B). This should influence therapeutic management.

The therapeutic approach to J-LN patients is complicated by the early age at which most of these patients become symptomatic and especially by the fact that **β**-blockers appear to have only limited efficacy. Also left cardiac sympathetic denervation (LCSD), employed in only a few patients, does not appear as effective in other LQTS patients. These data suggest that for many J-LN patients an implantable cardioverter defibrillator (ICD) should be seriously considered, in addition to the traditional therapies. For the subgroups at lower risk, as described above, it may be logical to postpone a decision to implant an ICD until age 8–10.

## Etiology

Following the identification, in 1995 and 1996, of the first three LQTS genes associated with the most frequently encountered LQTS variants called respectively LQT1, LQT2, and LQT3, there has been a flourishing of identifications of genes proven or just thought to be associated with LQTS [28-30]. This includes the genes for LQT4 through LQT10 (Table 1).

Unfortunately, haste or enthusiasm has created a terminology problem because not all of these genes can be truly regarded as responsible for LQTS. Specifically, we consider that what have been called LQT4 [31] and LQT7 [32] (Andersen-Tawil syndrome) are complex clinical disorders in which a more or less modest prolongation of the QT interval – especially in the Andersen-Tawil syndrome [33] – is only a secondary epiphenomenon and should not be regarded as part of LQTS. LQT5, LQT6 and LQT8, although rare, are certainly part of LQTS. For LQT9 and LQT10, there are only preliminary descriptions and at this time should be regarded as putative forms of LQTS.

**Table 1:** Long QT syndrome (LQTS) subtypes, disease-associated genes and prevalence

**LQTS subtypes**	**Gene**	**Prevalence (% of all ** **genotyped cases)**
LQT1 and JLN1 (AR)	*KCNQ1*	>50
LQT2	*KCNH2*	35–40
LQT3	*SCN5A*	10–15
LQT4	*ANK2*	
LQT5 (RWS) and JLN2	*KCNE1*	
LQT6	*KCNE2*	
LQT7 (Andersen-Tawil syndrome)*	*KCNJ2*	
LQT8 (Timothy syndrome)	*CACNA1c*	
LQT9	*CAV3*	
LQT10	*SCN4B*	

Abbreviations: LQTS = Long QT syndrome; JLN = Jervell and Lange-Nielsen syndrome; RWS = Romano-Ward syndrome; AR = autosomal recessive

* We consider the Andersen-Tawil syndrome to be a disorder different from LQTS (see ref. [78])

The main characteristic of all the LQTS genes identified so far is to encode cardiac ion channel subunits or, as in the case of the newly described *CAV3*, to modulate ionic currents: hence, the use of the word "channelopathy" to indicate the group of diseases to which LQTS belongs.

### *KCNQ1 *(LQT1) and *KCNE1 *(LQT5)

The delayed rectifier current (I_K_) is a major determinant of the phase 3 of the cardiac action potential. It comprises two independent components: one rapid (I_Kr_) and one slow, (I_Ks_).

The *KCNQ1 *gene and the *KCNE1 *gene encode respectively the alpha (*KvLQT1*) and the **β **(MinK) subunit of the potassium channel conducting the I_Ks _current. *KCNQ1 *mutations are found in the LQT1 variant of LQTS which is also its most prevalent form. Approximately half of the genotyped patients carry a mutation on this gene.

Homozygous or compound heterozygous mutations of *KCNQ1 *have been associated with the recessive Jervell and Lange-Nielsen form of LQTS (JLN1). LQT5 is an uncommon variant of LQTS caused by mutations in the *KCNE1 *gene: it accounts for approximately 2–3% of all genotyped LQTS patients. Mutations in the *KCNE1 *gene cause both Romano-Ward (LQT5) and Jervell and Lange-Nielsen (JLN2) syndromes.

### *KCNH2 *(LQT2) and *KCNE2 *(LQT6)

The *KCNH2 *gene and the *KCNE2 *gene encode respectively the alpha (*HERG *– Human Ether-a-go-go Related Gene) and the **β **(MIRP) subunit of the potassium channel conducting the I_Kr _current. This is the second most common variant of LQTS accounting for 35–40% of mutations in LQTS genotyped patients. Functional expression studies have demonstrated that mutations in the *KCNH2 *gene cause a reduction of I_Kr _current. *KCNH2 *mutants have a reduced function compared to the wild type peptides, therefore I_Kr _channels that incorporate mutated subunits carry a reduced I_Kr _repolarizing current. Defective proteins may either cause a dominant negative effect on the wild-type subunits or they may not interfere with the function of the normal subunits thus causing haploinsufficiency. Trafficking abnormalities have also been reported as a consequence of *KCNH2 *mutations [34].

Mutations in the *KCNE2 *gene are found in the LQT6 variant of LQTS. This gene encodes MiRP1 (MinK Related Peptide 1), a small peptide that coassembles with the HERG protein to form the I_Kr _channel. There are only few examples of *KCNE2 *mutations associated with LQTS.

### *SCN5A *(LQT3) and *SCN4B *(LQT10)

The *SCN5A *gene encodes the protein of the cardiac sodium channel. The Na^+ ^channel protein is a relatively large molecule that folds onto itself to surround the channel pore. The first *SCN5A *mutations identified were clustered in the regions that regulate the inactivation of the channel (**Δ**KPQ, R1623Q, N1325S). *In-vitro *expression studies [35] showed that these mutations cause an increased late inward sodium current (I_Na_). It was concluded that Na^+ ^channel mutations produce the LQTS phenotype by inducing a "gain of function" leading to increase in the Na^+ ^inward current which prolongs action potential duration. The prevalence of LQT3 among LQTS patients is estimated to be 10–15%.

Recently, a mutation on the sodium channel subunit NaV **β**4 was identified in an asymptomatic child with major QT prolongation and 2:1 AV block [36]. The mutation (L179F) leads to an increased window current with a molecular phenotype consistent with LQT3. This might become LQT10.

### *CACNA1c *(LQT8) – Timothy syndrome

LQT8 is a rare variant of LQTS characterized by marked QT interval prolongation, often presenting with 2:1 functional atrioventricular block and macroscopic T wave alternans, and syndactyly. LQT8 is highly malignant, and 10/17 (59%) of the children reported by Splawski *et al*. [37] died at a mean age of 2.5 years. Some children with Timothy syndrome also had congenital heart diseases, immune deficiency, intermittent hypoglycemia, cognitive abnormalities, and autism.

Molecular screening identified two missense mutation in the voltage-gated calcium channel gene (*CACNA1c*), in all probands analyzed [37], causing a reduced channel inactivation responsible for calcium overload, a well known mechanism for tissue damage and arrhythmias induction.

### *CAV3 *(LQT9)

The *CAV3 *gene encodes the caveoline 3, a protein component of caveolae and an integral membrane protein in trans-Golgi-derived vesicles. Caveolins participate in many important cellular processes, including vesicular transport and signal transduction.

Four missense mutations in *CAV3*-encoded caveoline 3 were recently identified in four LQTS probands and in none of the 400 reference alleles [38]. All the mutations identified (T78M, F97C, K30R and L86P) produced a gain-of-function, LQT3-like molecular/cellular phenotype, when expressed in human embryonic kidney (HEK) cells stably expressing the *SCN5A*-encoded cardiac sodium channel. *CAV3 *mutations were also implicated in a few sudden infant death syndrome cases [39,40].

## Diagnosis

### Clinical diagnosis

Given the characteristic features of LQTS, the typical cases present no diagnostic difficulty for the physicians aware of the disease. However, borderline cases are more complex and require the evaluation of multiple variables besides clinical history and surface electrocardiogram. To overcome these difficulties, diagnostic criteria were first proposed in 1985 [22] and were subsequently updated in 1993 [41] and then again in 2006 [42].

The new diagnostic criteria are listed in Table 2, with relative points assigned to various electrocardiographic, clinical, and familial findings. The score ranges from a minimum value of 0 and a maximum value of 9 points. Based on our experience, we have arbitrarily divided the point score into three probability categories: 1) ≤ 1 point = low probability of LQTS; 2) > 1 to 3.0 points = intermediate probability of LQTS; and 3) ≥ 3.5 points = high probability of LQTS. As QTc overcorrects at fast heart rates, additional diagnostic caution is necessary when dealing with a patient with tachycardia or with infants.

**Table 2:** 1993–2006 Long QT syndrome (LQTS) diagnostic criteria

			**POINTS**
**ELECTROCARDIOGRAPHIC FINDINGS #**			
A	QTc^	> 480 ms	3
		460 – 470 ms	2
		450 – 459 (male) ms	1
B	TORSADE DE POINTES *		2
C	T WAVE ALTERNANS		1
D	NOTCHED T WAVE IN 3 LEADS		1
E	LOW HEART RATE FOR AGE @		0.5
**CLINICAL HISTORY**			
A	SYNCOPE *	WITH STRESS	2
		WITHOUT STRESS	1
B	CONGENITAL DEAFNESS		0.5
**FAMILY HISTORY $**			
A	FAMILY MEMBERS WITH DEFINITE LQTS		1
B	UNEXPLAINED SUDDEN CARDIAC DEATH BELOW AGE 30 AMONG IMMEDIATE FAMILY MEMBERS		0.5

# In the absence of medications or disorders known to affect these electrocardiographic features

^ QTc calculated by Bazett's formula where QTc = QT/√RR

* Mutually exclusive

@ Resting heart rate below the 2^nd ^percentile for age

$ The same family member cannot be counted in A and B

**SCORE**: ≤ 1 point = low probability of LQTS

> 1 to 3 points = intermediate probability of LQTS

≥ 3.5 points = high probability of LQTS

These diagnostic criteria were conceived in the pre-molecular era and should be used with common sense. Obviously, they cannot be of value in identifying the so called "silent mutation carriers" who have a normal QT interval. For these individuals, molecular screening is essential. The main value of these clinical criteria is during a first contact with a patient (when one attempts to verify the likelihood of the presence of LQTS with potential therapeutic decisions while molecular diagnosis could not be available for at least a few months) and in clinical studies when a certain degree of uniformity in diagnosis is essential.

### Molecular diagnosis

#### Who should be screened for LQTS

Molecular diagnosis should always be attempted in families or individuals in whom the diagnosis of LQTS has either been made or is suspected on sound clinical grounds. When molecular diagnosis is successful (70–80% of cases in our laboratory), it does conclusively establish the disease state in clinically borderline individuals and especially in apparently unaffected individuals.

As discussed earlier, the penetrance of the disease can be low, therefore it is essential always trying to genotype the proband in a LQTS family. Successful genotyping will allow rapid screening of all family members and identification of 10–35% of mutation carriers who may have a normal QT interval, but may be anyhow at risk of life-threatening arrhythmias if not appropriately diagnosed and treated.

#### Molecular genetics and risk stratification

Molecular genetics contribute importantly to risk stratification. In 1998 Zareba *et al*. [43] suggested that LQT1 and LQT2 patients had more events than LQT3 patients but that the events in the latter group had greater lethality. These data were limited in part by the fact that they were based on 38 families, with the possibility of excessive weight by certain families. In 2003 Priori *et al*. [13] published data on a truly representative population (647 patients of known genotype coming from 193 families). The incidence of life-threatening events was lower among LQT1 patients compared to the other genotypes, partly because of the high prevalence of silent mutation carriers (QTc < 440 ms); the risk was higher among LQT2 females *vs*. males and LQT3 males *vs*. females. An important point is that, independently of genotype, the quartiles of QTc (<446 ms, between 447 and 468 ms; between 469 and 498 ms; >498 ms) provide very useful information concerning risk of becoming symptomatic. It also became evident that among LQT1 carriers many (37%) are silent mutation carriers and that the majority of LQT1 patients goes through life without ever suffering cardiac events. Despite a more serious clinical pattern, it has to be noted that also among LQT2 and LQT3 patients almost half remains asymptomatic; this fact is often forgotten as dramatically shown by the growing attitude, especially in the US, to implant ICDs in asymptomatic individuals just because they have been diagnosed as affected by LQTS.

Risk stratification guided by molecular genetics represents a very active area of research and significant progress is continuously being made. Donger *et al*. [43] have the merit of having been the first to go beyond "the gene" and to consider topology. In 1997 they suggested that mutations located in the C-terminal region might be associated with a less severe clinical phenotype, a suggestion renewed in 2001 by Piippo *et al*. [44]. In 2002 Moss *et al*. [45] indicated that LQT2 patients with mutations in the pore region of KCNH2 were at higher risk compared with patients with mutations in different region of the same gene. In 2007 Moss *et al*. [46] demonstrated in 600 LQT1 patients that both the transmembrane location of the mutations and their dominant-negative effect are independent risk factors for cardiac events. Shortly afterwards, still in 2007 Crotti *et al*. [47] carried one step further the quest for genetic markers contributing to risk stratification. In a collaborative study focusing on the hot spot *KCNQ1*-A341V (a common mutation present worldwide and responsible for a major founder effect in almost 25 South African families), they have demonstrated that the unusually high clinical severity already reported by Brink *et al*. [24] for the South African families is present also among LQT1 patients from different ethnic backgrounds but carrying the same A341V mutation. Moreover, as *KCNQ1*-A341V has a mild dominant-negative effect (the current loss barely exceeds 50%) its striking clinically severe phenotype is explained neither by the location (transmembrane) nor by the functionally consequence of the mutation (dominant-negative). This implies that the current biophysical assessments of the electrophysiological effects of LQTS-causing mutations do not provide all the information necessary to make a complete genotype-phenotype correlation. In this regard, the study by Crotti *et al*. [47] paves the way toward a mutation-specific risk stratification. In a near future, at least for certain cases, it will become possible to implement a management specifically directed to provide the best protection to LQTS patients on the basis of their individual mutation.

Mutations located in the pore region of the protein are usually associated with a more severe clinical phenotype compared to mutation in the C-terminal region [45]. Unfortunately, the risk stratification process is not simple, as additionally genetic variants may be present and may modify clinical severity. An example is provided by an LQT2 family with the C-terminal A1116V mutation. The proband who had a severe form of the disease at variance with her family-members all asymptomatic, was also the only member of the family carrying the very common *KCNH2*-K897T polymorphism, and genetic and electrophysiological evidence indicates that K897T produces an accentuation of the mutation-dependent I_Kr _current loss resulting in the unmasking of a clinically latent C-terminal LQT2 mutation [48,49].

## Relation with sudden infant deaths

Sudden infant death syndrome (SIDS) remains the leading cause of sudden death during the first year of life in the western world. Despite a large number of theories mostly focused on abnormalities in the control of respiratory or cardiac function [50], the causes of SIDS remain largely unknown. In 1976 Schwartz proposed that an undefined number of SIDS victims might die because of an arrhythmic death favored by a prolongation of the QT interval with a mechanism similar to that of LQTS [51]. Few months later Maron *et al*. also suggested that LQTS could contribute to SIDS [52].

This hypothesis was tested by prospectively measuring the QT interval during the first week of life in more than 33,000 infants and by following them for a possible occurrence of SIDS [53]. There were 34 deaths, of which 24 were due to SIDS. The infants who died of SIDS had a longer QTc (measured blindly to the outcome) than the survivors and the victims from other causes. Moreover, 12 of the 24 SIDS victims but none of the other dead infants had a prolonged QTc (defined *a priori *as exceeding 440 ms). The odds ratio for SIDS for infants with a prolonged QTc was 41, reaching 47 for male infants. The unavoidable conclusion of that study was that a QT prolongation in the first week of life represents a major risk factor for SIDS.

Subsequently, two proof-of-concept identifications of LQTS-causing mutations in a victim of SIDS [54] and in an infant who survived a typical episode of near-miss episode with documented ventricular fibrillation [55] paved the way to two cohort studies. The first one found LQTS-causing mutations in 5.2% of 68 white infants [56]. The second [40], based on 201 SIDS victims and 187 controls, all from Norway, identified functional [57,58] mutations in LQTS genes in 9.5% (95% confidence intervals, 5.8 – 14.4) of the victims and in none of the controls. Considering that in 30% of unequivocal cases of LQTS no mutations are found, it is likely that 11–13% of cases currently labeled as SIDS are actually due to LQTS. As such, these are preventable deaths.

These data obviously support the controversial concept of neonatal ECG screening [59,60] according to the guidelines proposed by the Task Force of the European Society of Cardiology [10]. The aim is the prevention of those sudden deaths due to unrecognized LQTS which may occur during the first few months of life or later on in life. Importantly, such a screening is markedly cost-effective in Europe [61].

### Differential diagnosis

Typical cases of LQTS are so characteristic that differential diagnosis is not even considered. When dealing with borderline cases, the following conditions should be considered: vasovagal syncope, orthostatic hypotension, arrhythmogenic right ventricular cardiomyopathy/dysplasia (ARVC/D), catecholaminergic polymorphic ventricular tachycardia (CPVT), hypertrophic cardiomyopathy, ventricular tachycardia, drug-induced long QT syndrome, epilepsy.

## Management including treatment

The trigger for most of the episodes of life-threatening arrhythmias of LQTS is represented by a sudden increase in sympathetic activity, largely mediated by the quantitatively dominant left cardiac sympathetic nerves [22]. Indeed, antiadrenergic therapies provide the greatest degree of protection. However, some patients have syncopal episodes while being asleep or at rest, or when they suddenly aroused from these states, and in some cases the arrhythmias are pause-dependent. As discussed above, the triggering conditions are largely gene-dependent [15].

### Antiadrenergic interventions

#### β-adrenergic blockade

**β**-adrenergic blocking agents represent the first choice therapy in symptomatic LQTS patients, unless specific contraindications are present.

Propranolol is still the most widely used drug, at a daily dosage of 2 to 3 mg/kg; sometime the dosage is increased to 4 mg/kg. The main advantages of propranolol are its lipophilicity that allows it to cross the blood-brain barrier, and its well known tolerability for chronic therapy. Its main disadvantages are the need of multiple daily administrations and the contraindications for patients with asthma and diabetes. For these reasons nowadays nadolol is used more frequently, as its longer half-life allows twice-a-day administration, usually at 1 mg/kg/day. Atenolol has been reported to be associated with clinical failures more often than propranolol or nadolol. **β**-blockers rarely result in excessive bradycardia, especially if the dosage is very gradually increased over several weeks.

In a large number of patients of unknown genotype, mortality on **β**-blocker therapy was 2%, and it was 1.6% when limited to patients with syncope (no cardiac arrest) and without events in the first year of life [62]. There is clear evidence that **β**-blockers are extremely effective in LQT1 patients. Data from two large studies [63,64] indicate that mortality is around 0.5% and sudden death combined with cardiac arrest reaches 1%. The impairment in the I_Ks _current makes these patients particularly sensitive to catecholamines and quite responsive to **β**-blockade. These patients seldom need more than antiadrenergic therapy.

Compared to LQT1 patients, LQT2 patients have more life-threatening events despite **β**-blockers, but most of these are resuscitated cardiac arrest (6–7%) [63]. Among LQT3 patients major events occur more frequently (10–15%) despite **β**-blockers [15,63], and several of these patients require additional therapies (see below). Also many patients with Jervell and Lange-Nielsen syndrome are not adequately protected by **β**-blockers [3].

Compliance is essential for LQTS patients treated with **β**-blockers. Most of the so-called failures of **β**-blockers therapy are due to incomplete compliance [64].

#### Left cardiac sympathetic denervation (LCSD)

Following a small incision in the left subclavicular region, LCSD is performed by an extrapleural approach which makes thoracotomy unnecessary. The average time for the complete operation is 35–40 minutes [65]. LCSD requires removal of the first four thoracic ganglia. There is no need to ablate the cephalic portion of the left stellate ganglion. In this way the Horner's syndrome (ptosis and miosis) can almost always be avoided [65]. In approximately 30% of patients there is a very modest (1–2 mm) ptosis which can be noted only by close examination but fully escapes notice in normal social interactions.

**Figure 5:**
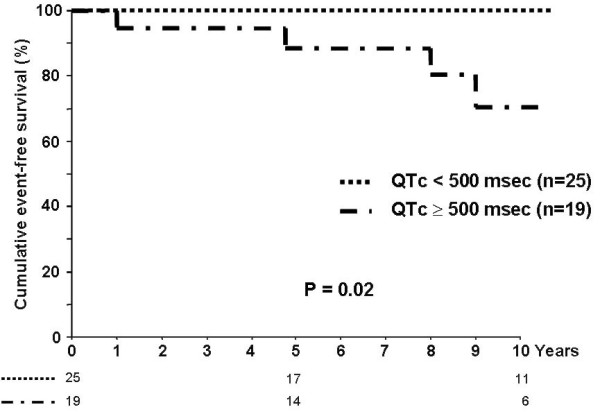
**Kaplan-Meier curves of event-free survival and survival according to QTc interval after left cardiac sympathetic denervation in patients with only syncope, or aborted cardiac arrest before left cardiac sympathetic denervation**. (*Modified from ref*. [65]* with permission from Lippincott Williams and Wilkins)*.

The data published in 1991 [66] were updated in 2004 to include 147 LQTS patients who underwent LCSD during the last 35 years [65]. These patients constituted a very high risk group, as witnessed by the fact that 99% were symptomatic, that their mean QTc was very long (563± 65 ms), that 48% had a cardiac arrest and especially that 75% continued to have syncope despite full-dose **β**-blockers. The data most relevant to clinical decisions nowadays are those regarding patients without cardiac arrest (who currently properly receive an ICD) who suffer syncope despite being treated with a full dose of **β**-blockers. During a mean follow-up of eight years there was a 91% reduction in cardiac events. LCSD was associated with a mean QTc shortening of 39 ms, pointing to an action on the substrate as well as on the trigger. Mortality was 3% in this high risk group. A post-surgery QTc < 500 ms predicted a very favorable outcome (Fig. 5). Practically of great relevance is the fact that this series included five patients who underwent LCSD due to multiple ICD shocks and electrical storms: in this group, over a 4-year follow-up there was a 95% decrease in the number of shocks (from an average of 29 shocks/year) with a dramatic improvement in the quality of life of the patients and of their families.

The major antifibrillatory efficacy of LCSD has been previously demonstrated in high-risk post-myocardial infarction patients [67], and very recently in patients with catecholaminergic polymorphic ventricular tachycardia not protected by **β**-blockers and receiving unnecessary shocks by the ICD [68].

Whenever syncopal episodes recur despite a full-dose **β**-blocking therapy, LCSD should be considered and implemented whenever possible.

### Cardiac pacing

The LQTS patients for whom cardiac pacing is indicated are very few. In infants or young children with 2:1 AV block this remains a reasonable choice, as a bridge to the ICD. The fact that the onset of TdP is very often preceded by a pause [14] might add to the rationale of using a pacemaker as an adjunct to the therapy of some LQTS patients. However, nowadays if a pacemaker is being considered, it is probably more logical to implant an ICD with pacing modes. In any case, pacemakers should never be used as a sole therapy for LQTS.

### Implantable cardioverter defibrillators (ICDs)

There has been a major increase, largely unjustified, in the number of ICDs implanted in LQTS patients. It is uniformly agreed that in case of a documented cardiac arrest, either on or off therapy, an ICD should immediately be implanted. By contrast, there are strongly different opinions regarding the use of ICDs in patients without cardiac arrest.

The available data from both the US [69] and the European [70] ICD-LQTS Registries provide the disquieting information that the majority of implanted patients had not suffered a cardiac arrest and, moreover, that many had not even failed **β**-blocker therapy. A recent multicenter study went as far as indicating that in their protocol the mere presence of a *SCN5A *mutation, even in a totally asymptomatic individual, is sufficient for immediate ICD implant; the authors added that none of their LQT3 patients has received shocks so far [71].

It should not be forgotten that the implantable defibrillator does not prevent occurrence of malignant arrhythmias and that TdP are frequently self-terminating in LQTS, as indicated by the high incidence of syncopal episodes with spontaneous recovery. The recurrence of electrical storms has led to suicidal attempts in teenagers and its high incidence (>10%) in children has been considered by a large group of US pediatric cardiologists as "devastating" [72]. The massive release of catecholamines, triggered by pain and fear, that follows an ICD discharge in a conscious patient – especially a young one – leads to further arrhythmias and to further discharges which produce a dramatic vicious circle. To overcome these limitations we have designed a new algorithm for ICDs specifically tailored for the young LQTS patients that is currently under clinical evaluation. This includes, prior to discharge, sufficient time to allow a spontaneous return to sinus rhythm after onset of Torsade-de-Pointes/ventricular fibrillation and to then automatically institute a period of relatively rapid pacing to prevent reinitiation of life-threatening arrhythmias and new shocks.

Special caution is necessary before choosing to implant an ICD in a child with LQTS. Such a decision is seldom justified before a proper trial with combined antiadrenergic therapy (*i.e*. full-dose **β**-blockade and LCSD), which prevents sudden death in 96–97% of symptomatic and high-risk patients [65] and which still allow an ICD implant in case of a new syncope or cardiac arrest. On the other hand, the nature of the disease is such that cardiac arrests may recur and have lethal consequences. Thus, concerns for the life of the patients and the interference of medico-legal considerations represent a reality. The risk-benefit ratio of an ICD should be clearly explained to the patient or to his/her parents together with information on the pro and cons of LCSD, to allow them the possibility of a choice. Paradoxically, the decision to implant an ICD as an additional precaution even in a patient who appears to do well with combined antiadrenergic therapy, has a certain logic because such a patient has a low but not zero risk of cardiac arrest. This makes cardioversions unlikely to occur but the presence of the ICD may provide a means of survival against the unexpected episode of an otherwise lethal arrhythmia. The need for several battery and leads replacements remains a major problem with young patients.

Our current policy is to implant an ICD always after a cardiac arrest, always when requested by the patient, and whenever syncope recurs despite **β**-blockade and LCSD.

The long QT syndrome is one of the leading cause of sudden cardiac death in young otherwise healthy individuals and contributes significantly to SIDS. As very effective therapies do exist, it is important to make a very early diagnosis, *i.e*. with a program of neonatal ECG screening, to be able to act in a preventive manner.

To make a correct diagnosis various electrocardiographic, clinical, and familial findings must be taken into account and specific diagnostic criteria have been developed (Table 2). Additionally, molecular screening in LQTS is now part of the diagnostic process, as in 70% of the affected cases a mutation in the already known LQTS genes can be identified.

The identification of the genetic defect responsible for the disease in a family is also important allowing to identify all affected family members at risk for life-threatening cardiac arrhythmias, even those with a normal QTc (silent mutation carriers). A proper example is that of a family with a boy and a girl, the boy and one parent being clearly affected (syncope and prolonged QTc). If the girl has a normal QT interval and is incorrectly assumed to be unaffected, she might later on be treated with one of the many drugs that block the I_Kr _current, and could develop TdP and die. This would not be a bad luck; it would be the direct responsibility of the physician who had not attempted molecular screening in the mother or boy who were clearly affected. Identification of the disease-causing mutation would have allowed to rapidly establish beyond doubt if the girl was or not a mutation carrier and this would have saved her life.

The identification of the disease causing mutation is also helpful in the risk stratification process, as not only the gene [15] but also the specific site of the mutation [43-46] and sometimes the specific mutation [24,47] can be associated with a different risk for cardiac arrhythmias and different response to therapy. Therefore, the genetic data is very useful for the correct management of these patients. As a matter of fact, according to the genotype, different is the response to **β**-blocker therapy (very good in LQT1, reasonable in LQT2 and apparently poor in LQT3) and also to conditions that could favor the occurrence of life-threatening arrhythmias are different. LQT1 patients are at higher risk during sympathetic activation, such as during exercise and emotions. They should not be allowed to participate in competitive sports. Swimming is particularly dangerous, as 99% of the arrhythmic episodes associated with swimming occur in LQT1 patients [15].

In LQT2 patients, some of whom have a tendency to lose potassium, it is essential to preserve adequate potassium levels. Oral K^+ ^supplements in combination with K^+ ^sparing agents are a reasonable approach. As these patients are at higher risk especially when aroused from sleep or rest by a sudden noise [15,73], we recommend that telephones and alarm clocks are removed from their bedrooms and, especially with children, whenever they have to be wakened up in the morning, to do it gently and without yelling. These rather gross management measures actually constitute a form of gene-specific therapy.

**Figure 6:**
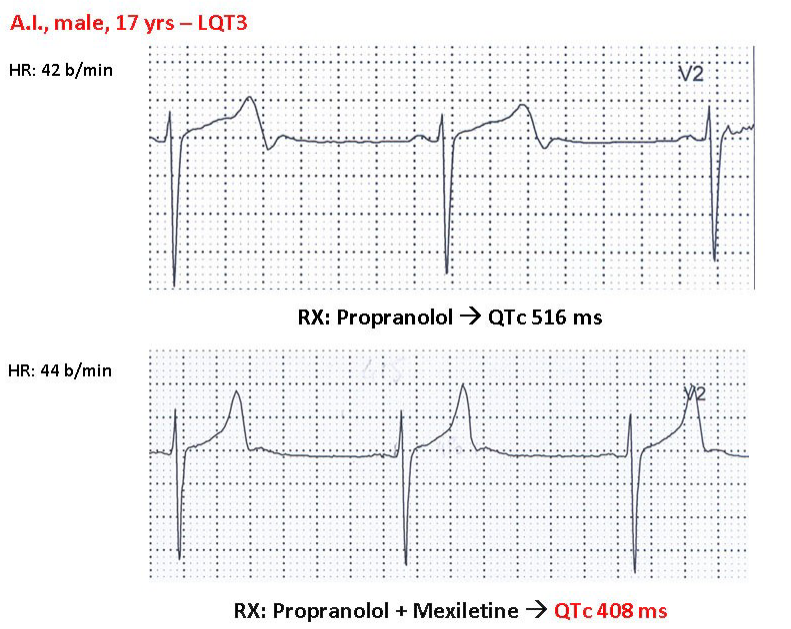
**LQT3 patient aged 17-yrs, major QTc prolongation (QTc 516 ms) not modified by **β**-blocker therapy.** The addition of oral chronic therapy with mexiletine (200 mg b.i.d.) produced a major shortening of QTc by over 100 ms persisting over time. (*Reprinted from ref*. [78]* with permission from Elsevier)*.

The realization that *SCN5A *mutations producing LQT3 have a "gain-of-function" effect [35] has lent support to our early suggestion [74] to test sodium channel blockers, and especially mexiletine, as possible adjuvants in the management of LQT3 patients [42,75-77]. Our current policy is to test the effectiveness of mexiletine in all LQT3 patients by the acute oral drug test technique, which requires administration of half the daily dose during continuous ECG monitoring. Within 90 minutes the peak plasma concentration is reached and if the QTc is shortened by more than 40 ms then we add mexiletine to the **β**-blocker therapy. As a matter of fact, in most LQT3 patients with mexiletine QTc shortens by more than 70–80 ms (Fig. 6)[78]. Even though there is no conclusive evidence for a beneficial effect and definite failures have occurred, there is also growing evidence of significant benefit in a number of individual cases. Not all patients carrying *SCN5A *mutations respond equally to mexiletine [42]. There are cases of highly malignant forms that manifest in infancy and are due to mutations causing a severe electrophysiological dysfunction that are corrected by both mexiletine and propranolol [42].

Interestingly, during heart rate increases, QTc shortens more in LQT3 patients than among healthy controls [74] and, indeed, normal physical activity may not need to be restricted in LQT3 patients. They are at higher risk of death at rest and especially nighttime. When these patients sleep and are in a horizontal position, the onset of TdP produces a progressive but slow fall in blood pressure that facilitates a noisy gasping preceding death. This has led both LQT2 (also at risk while resting) and LQT3 patients to be saved by a family member, such as the spouse sleeping in the same bed. Accordingly, we recommend that LQT2 and LQT3 patients have an intercom system in their and – if young – in their parents bedrooms.

Finally, QT interval shortening, albeit encouraging, cannot be assumed automatically to imply protection from life-threatening arrhythmias. One should never forget the tragic failures of the attempts to shorten QT by digitalis [1]. The use of novel and experimental therapies should not deprive symptomatic patients of therapies with established protective effect.

Given the risk of approximately 12% of sudden death as first manifestation of LQTS, we consider necessary to initiate **β**-blocker treatment in all patients, including those still asymptomatic. Among the asymptomatic patients, reasonable exceptions appear to be LQT1 males above age 20–25 because very rarely they become symptomatic above this age, and adults with a QTc < 500 ms. LQT2 women seem to remain at risk throughout life and we recommend to treat them always. Patients with a normal QTc (< 440 ms) appear to be at very low risk for life-threatening arrhythmias. In absence of clear follow-up data their treatment is optional; the characterization of the mutation could influence this choice.

Cardiac and non-cardiac drugs that block the I_Kr _current and thereby prolong the QT interval should always be avoided by LQTS patients. A drugs list is published at Arizona Center for Education and Research on Therapeutics [79]. Such a list, updated every year, should be given to all LQTS patients because their responsible physicians may not be aware of these electrophysiological actions.

## Conclusion

In conclusion, the sound data available, and decades of clinical experience, dictate the therapeutic approach to the patient affected by LQTS who already has had a syncopal episode. Treatment should always begin with **β**-blockers, unless there are valid contraindications. If the patient has one more syncope despite full dose **β**-blockade, LCSD should be performed without hesitation and ICD implant should be considered with the final decision being based on the individual patient characteristics (age, sex, previous history, genetic subgroup including sometime mutation-specific features, presence of ECG signs – including 24-hour Holter recordings – indicating high electrical instability). In the end, there is no substitute for careful clinical judgment, accompanied by a thorough knowledge of the several variants of this unique life-threatening cardiac disorder, when the goal is to protect the patients and at the same time to ensure their quality of life.

## Abbreviations

LQTS: Long QT syndrome; J-LN: Jervell and Lange-Nielsen syndrome; R-W: Romano-Ward syndrome; ECG: Electrocardiogam; QTc: Corrected QT; TdP: Torsade-de-Pointes; AV: Atrioventricular; LCSD: Left cardiac sympathetic denervation; ICD: Implantable cardioverter defibrillator; I_K_: Delayed rectifier current HERG: Human ether-a-go-go related gene; MiRP1: MinK Related Peptide 1; HEK: Human embryonic kidney; SIDS: Sudden infant death syndrome.

## Competing interests

The authors declare that they have no competing interests.

## Authors' contributions

All authors contributed to this review article. They read and approved the final version of the manuscript.
